# *tert*-Butyl 4-{[2-amino-4-(2-hy­droxy­phen­yl)pyrimidin-5-yl]meth­yl}piperazine-1-carboxyl­ate

**DOI:** 10.1107/S1600536813025774

**Published:** 2013-09-21

**Authors:** Nilesh N. Gajera, Mukesh C. Patel, Mukesh M. Jotani, Edward R. T. Tiekink

**Affiliations:** a P. S. Science and H. D. Patel Arts College, S. V. Campus, Kadi, Gujarat, 382 715, India; b Department of Physics, Bhavan’s Sheth R. A. College of Science, Ahmedabad, Gujarat, 380 001, India; c Department of Chemistry, University of Malaya, 50603 Kuala Lumpur, Malaysia

## Abstract

In the title compound, C_20_H_27_N_5_O_3_, the central piperazine ring adopts a chair conformation, with the N-bound carboxyl­ate and methyl­ene substituents occupying bis­ectional and equatorial orientations, respectively. A twist is evident between the aromatic rings [dihedral angle = 25.61 (9)°] but an intra­molecular O—H⋯N hydrogen bond persists between these. Supra­molecular tapes along [1-10] are formed in the crystal packing through N(amino)—H⋯O(hydrox­yl) and N(amino)—H⋯N(pyrimidin­yl) hydrogen bonds, and these are linked into layers in the *ab* plane by π–π inter­actions [inter-centroid distance between pyrimidinyl rings = 3.5919 (9) Å].

## Related literature

For the biological activity of pyrimidine-containing heterocyclic compounds, see: Topalis *et al.* (2011 ▶); Sbardella *et al.* (2011 ▶); Zhang *et al.* (2011 ▶). For the synthesis, see: Patel *et al.* (2011 ▶).
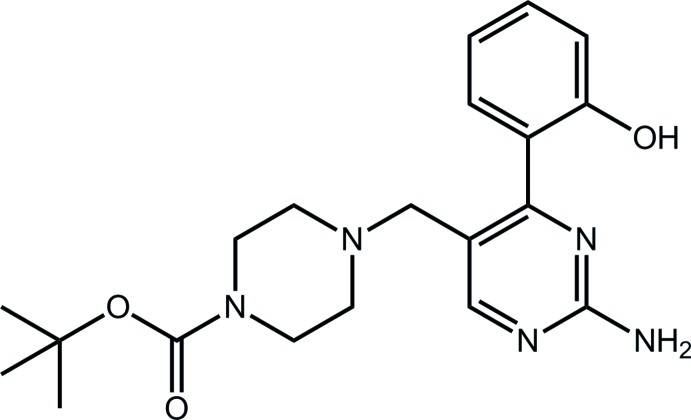


## Experimental

### 

#### Crystal data

C_20_H_27_N_5_O_3_
*M* *_r_* = 385.47
Monoclinic, 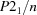

*a* = 6.1925 (2) Å
*b* = 8.2636 (2) Å
*c* = 40.7287 (11) Å
β = 93.513 (2)°
*V* = 2080.27 (10) Å^3^
*Z* = 4
Mo *K*α radiation
μ = 0.09 mm^−1^
*T* = 293 K
0.47 × 0.35 × 0.31 mm

#### Data collection

Bruker APEXII CCD diffractometer
Absorption correction: multi-scan (*SADABS*; Bruker, 2004 ▶) *T*_min_ = 0.980, *T*_max_ = 0.997
20251 measured reflections
4985 independent reflections
3339 reflections with *I* > 2σ(*I*)
*R*_int_ = 0.026

#### Refinement

*R*[*F* ^2^ > 2σ(*F* ^2^)] = 0.050
*wR*(*F* ^2^) = 0.141
*S* = 1.04
4984 reflections
265 parameters
3 restraints
H atoms treated by a mixture of independent and constrained refinement
Δρ_max_ = 0.19 e Å^−3^
Δρ_min_ = −0.17 e Å^−3^

### 

Data collection: *APEX2* (Bruker, 2004 ▶); cell refinement: *APEX2* and *SAINT* (Bruker, 2004 ▶); data reduction: *SAINT*; program(s) used to solve structure: *SHELXS97* (Sheldrick, 2008 ▶); program(s) used to refine structure: *SHELXL97* (Sheldrick, 2008 ▶); molecular graphics: *ORTEP-3 for Windows* (Farrugia, 2012 ▶) and *DIAMOND* (Brandenburg, 2006 ▶); software used to prepare material for publication: *publCIF* (Westrip, 2010 ▶).

## Supplementary Material

Crystal structure: contains datablock(s) general, I. DOI: 10.1107/S1600536813025774/hg5347sup1.cif

Structure factors: contains datablock(s) I. DOI: 10.1107/S1600536813025774/hg5347Isup2.hkl

Supplementary material file. DOI: 10.1107/S1600536813025774/hg5347Isup3.cml

Additional supplementary materials:  crystallographic information; 3D view; checkCIF report

## Figures and Tables

**Table 1:** Hydrogen-bond geometry (Å, °)

*D*—H⋯*A*	*D*—H	H⋯*A*	*D*⋯*A*	*D*—H⋯*A*
O3—H1*O*⋯N4	0.83 (1)	1.80 (2)	2.560 (2)	151 (3)
N5—H1*N*⋯N3^i^	0.86 (1)	2.19 (1)	3.054 (2)	179 (2)
N5—H2*N*⋯O3^ii^	0.86 (1)	2.29 (1)	3.139 (2)	169 (2)

Symmetry codes: (i) 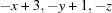; (ii) 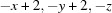.

**Fig. 1.**
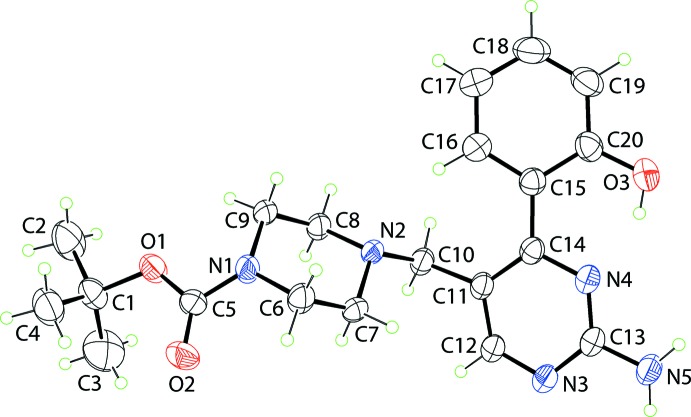
The molecular structure of (I) showing the atom-labelling scheme and displacement ellipsoids at the 35% probability level.

**Fig. 2.**
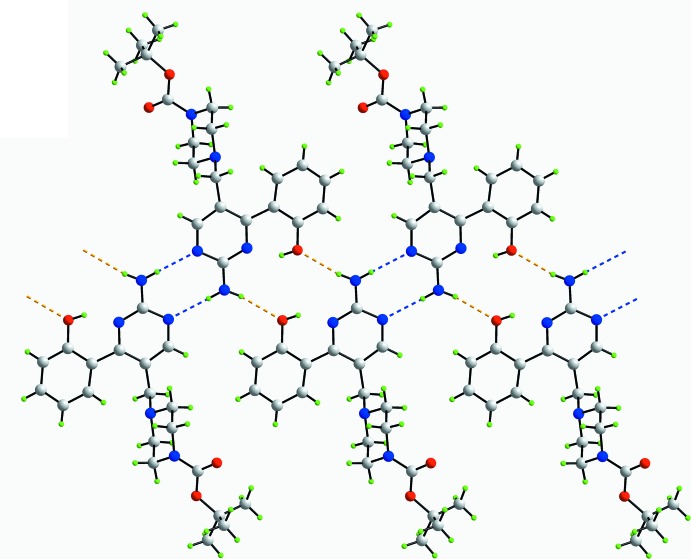
View of the supramolecular tape along [1 - 1 0] in (I) mediated by N—H···O, N hydrogen bonds, shown as orange and blue dashed lines, respectively.

**Fig. 3.**
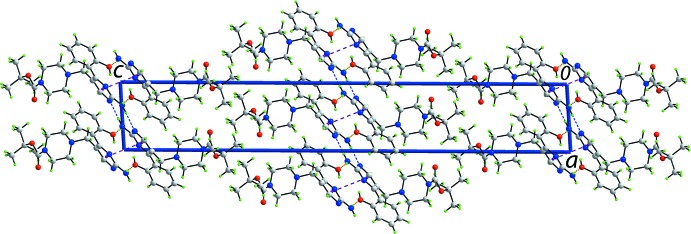
A view in projection down the b axis of the unit-cell contents for (I). The N—H···O, N—H···N and π—π interactions are shown as orange, blue and purple dashed lines, respectively.

## supplementary crystallographic information

### 1. Comment

Pyrimidine-containing heterocyclic compounds show significant biological importance through various activities such as anti-viral, anti-tumour, anti-proliferative, anti-diabatic, anti-bacterial, *etc.* (Topalis *et al.*, 2011; Sbardella *et al.*, 2011; Zhang *et al.*, 2011). It was the effectiveness of these derivatives against anti-microbial strains, such as S. aureus and P. aeruginosa, that motivated the present structural studies of a title compound, (I).

Despite there being a twist between the two aromatic rings in (I), Fig. 1, manifested in a dihedral angle of 25.61 (9)°, an intramolecular O—H···N hydrogen bond persists, Table 1. The piperazine ring adopts a chair conformation, and the N1- and N2-bound carboxylate and methylene substituents have bisectional and equatorial orientations, respectively.

In the crystal packing molecules assemble into supramolecular tapes along [1 - 1 0]. The amino-H atoms form hydrogen bonds (Table 1) to pyrimidinyl-N and hydroxyl-O atoms from different molecules, forming centrosymmetric eight-membered {···HNCN}_2_ and 16-membered {···HNCNC_3_O}_2_ synthons, respectively, Fig. 2. Layers in the *ab* plane are formed by π—π interactions occurring between centrosymmetrically related pyrimidinyl ring [inter-centroid distance = 3.5919 (9) Å for symmetry operation 2 - *x*, 1 - *y*, -*z*], with the layers being associated *via* hydrophobic interactions, Fig. 3.

### 2. Experimental

The title compound, (I), was prepared according to the procedure reported in the literature (Patel *et al.*, 2011). Crystals were obtained by were obtained by slow evaporation from an ethanol solution of (I).

### 3. Refinement

The C-bound H atoms were geometrically placed (C—H = 0.93–0.97 Å) and refined as riding with *U_iso_*(H) = 1.2–1.5*U_eq_*(C). The O– and N-bound H-atoms were refined with the distance restraints O—H = 0.82±0.01 and N—H = 0.86±0.01 Å, and with *U_iso_*(H) = 1.5*U_eq_*(O) and 1.2*U_eq_*(N).

### Crystal data

**Table:**

C_20_H_27_N_5_O_3_	*F*(000) = 824
*M**_r_* = 385.47	*D*_x_ = 1.231 Mg m^−^^3^
Monoclinic, *P*2_1_/*n*	Mo *K*α radiation, λ = 0.71073 Å
Hall symbol: -P 2yn	Cell parameters from 5662 reflections
*a* = 6.1925 (2) Å	θ = 2.5–24.9°
*b* = 8.2636 (2) Å	µ = 0.09 mm^−^^1^
*c* = 40.7287 (11) Å	*T* = 293 K
β = 93.513 (2)°	Block, yellow
*V* = 2080.27 (10) Å^3^	0.47 × 0.35 × 0.31 mm
*Z* = 4	

### Data collection

**Table:**

Bruker APEXII CCD diffractometer	4985 independent reflections
Radiation source: fine-focus sealed tube	3339 reflections with *I* > 2σ(*I*)
Graphite monochromator	*R*_int_ = 0.026
Detector resolution: 10.0 pixels mm^-1^	θ_max_ = 28.1°, θ_min_ = 1.0°
ω and φ scan	*h* = −8→8
Absorption correction: multi-scan (*SADABS*; Bruker, 2004)	*k* = −10→10
*T*_min_ = 0.980, *T*_max_ = 0.997	*l* = −53→53
20251 measured reflections	

### Refinement

**Table:**

Refinement on *F*^2^	Primary atom site location: structure-invariant direct methods
Least-squares matrix: full	Secondary atom site location: difference Fourier map
*R*[*F*^2^ > 2σ(*F*^2^)] = 0.050	Hydrogen site location: inferred from neighbouring sites
*wR*(*F*^2^) = 0.141	H atoms treated by a mixture of independent and constrained refinement
*S* = 1.04	*w* = 1/[σ^2^(*F*_o_^2^) + (0.0598*P*)^2^ + 0.4709*P*] where *P* = (*F*_o_^2^ + 2*F*_c_^2^)/3
4984 reflections	(Δ/σ)_max_ < 0.001
265 parameters	Δρ_max_ = 0.19 e Å^−^^3^
3 restraints	Δρ_min_ = −0.17 e Å^−^^3^

### Special details

**Table:**

Geometry. All s.u.'s (except the s.u. in the dihedral angle between two l.s. planes) are estimated using the full covariance matrix. The cell s.u.'s are taken into account individually in the estimation of s.u.'s in distances, angles and torsion angles; correlations between s.u.'s in cell parameters are only used when they are defined by crystal symmetry. An approximate (isotropic) treatment of cell s.u.'s is used for estimating s.u.'s involving l.s. planes.
Refinement. Refinement of *F*^2^ against ALL reflections. The weighted *R*-factor *wR* and goodness of fit *S* are based on *F*^2^, conventional *R*-factors *R* are based on *F*, with *F* set to zero for negative *F*^2^. The threshold expression of *F*^2^ > 2σ(*F*^2^) is used only for calculating *R*-factors(gt) *etc*. and is not relevant to the choice of reflections for refinement. *R*-factors based on *F*^2^ are statistically about twice as large as those based on *F*, and *R*- factors based on ALL data will be even larger.

### Fractional atomic coordinates and isotropic or equivalent isotropic displacement parameters (Å^2^)

**Table:**

	*x*	*y*	*z*	*U*_iso_*/*U*_eq_	
O1	0.91283 (18)	0.00416 (15)	0.20530 (3)	0.0595 (3)	
O2	1.2654 (2)	0.03360 (18)	0.19456 (4)	0.0730 (4)	
O3	0.7761 (2)	0.93303 (17)	0.02308 (4)	0.0754 (4)	
H1O	0.890 (3)	0.881 (3)	0.0213 (7)	0.113*	
N1	1.0148 (2)	0.2184 (2)	0.17776 (4)	0.0570 (4)	
N2	0.89391 (19)	0.36533 (16)	0.11658 (3)	0.0439 (3)	
N3	1.2749 (2)	0.47841 (18)	0.02609 (3)	0.0547 (4)	
N4	1.0670 (2)	0.71577 (17)	0.03355 (3)	0.0485 (3)	
N5	1.3458 (3)	0.7126 (2)	−0.00122 (4)	0.0586 (4)	
H1N	1.453 (2)	0.660 (2)	−0.0085 (5)	0.070*	
H2N	1.313 (3)	0.8128 (13)	−0.0043 (5)	0.070*	
C1	0.9389 (3)	−0.1517 (2)	0.22215 (5)	0.0643 (5)	
C2	0.7106 (4)	−0.1849 (3)	0.23163 (7)	0.0982 (8)	
H2A	0.6168	−0.1958	0.2121	0.147*	
H2B	0.6619	−0.0968	0.2446	0.147*	
H2C	0.7084	−0.2833	0.2442	0.147*	
C3	1.0205 (6)	−0.2767 (3)	0.19926 (8)	0.1142 (10)	
H3A	0.9265	−0.2812	0.1796	0.171*	
H3B	1.0233	−0.3805	0.2099	0.171*	
H3C	1.1640	−0.2485	0.1936	0.171*	
C4	1.0877 (4)	−0.1339 (3)	0.25247 (6)	0.0862 (7)	
H4A	1.2309	−0.1087	0.2462	0.129*	
H4B	1.0909	−0.2334	0.2647	0.129*	
H4C	1.0365	−0.0483	0.2659	0.129*	
C5	1.0795 (3)	0.0788 (2)	0.19248 (4)	0.0502 (4)	
C6	1.1675 (3)	0.3157 (3)	0.16089 (4)	0.0583 (5)	
H6A	1.3126	0.2742	0.1656	0.070*	
H6B	1.1633	0.4263	0.1688	0.070*	
C7	1.1151 (2)	0.3135 (2)	0.12434 (4)	0.0505 (4)	
H7A	1.2135	0.3848	0.1136	0.061*	
H7B	1.1346	0.2048	0.1160	0.061*	
C8	0.7452 (2)	0.2609 (2)	0.13308 (4)	0.0539 (4)	
H8A	0.7585	0.1509	0.1251	0.065*	
H8B	0.5978	0.2965	0.1278	0.065*	
C9	0.7909 (3)	0.2634 (2)	0.16981 (4)	0.0571 (5)	
H9A	0.7641	0.3710	0.1782	0.069*	
H9B	0.6955	0.1883	0.1801	0.069*	
C10	0.8377 (3)	0.3662 (2)	0.08106 (4)	0.0501 (4)	
H10A	0.6881	0.3998	0.0774	0.060*	
H10B	0.8489	0.2566	0.0728	0.060*	
C11	0.9763 (3)	0.4747 (2)	0.06151 (4)	0.0461 (4)	
C12	1.1442 (3)	0.4048 (2)	0.04594 (4)	0.0523 (4)	
H12	1.1684	0.2951	0.0497	0.063*	
C13	1.2268 (3)	0.6347 (2)	0.02015 (4)	0.0482 (4)	
C14	0.9433 (2)	0.6398 (2)	0.05486 (4)	0.0452 (4)	
C15	0.7717 (3)	0.7411 (2)	0.06797 (4)	0.0496 (4)	
C16	0.6802 (3)	0.7041 (3)	0.09754 (5)	0.0661 (5)	
H16	0.7374	0.6190	0.1102	0.079*	
C17	0.5086 (4)	0.7892 (3)	0.10848 (6)	0.0808 (6)	
H17	0.4508	0.7621	0.1283	0.097*	
C18	0.4230 (4)	0.9149 (3)	0.08982 (6)	0.0800 (6)	
H18	0.3016	0.9694	0.0964	0.096*	
C19	0.5151 (3)	0.9602 (2)	0.06172 (6)	0.0712 (6)	
H19	0.4582	1.0475	0.0497	0.085*	
C20	0.6908 (3)	0.8788 (2)	0.05086 (5)	0.0567 (4)	

### Atomic displacement parameters (Å^2^)

**Table:**

	*U*^11^	*U*^22^	*U*^33^	*U*^12^	*U*^13^	*U*^23^
O1	0.0471 (6)	0.0574 (7)	0.0739 (8)	0.0036 (5)	0.0027 (6)	0.0193 (6)
O2	0.0452 (7)	0.0813 (9)	0.0922 (10)	0.0159 (7)	0.0017 (6)	0.0178 (8)
O3	0.0799 (10)	0.0616 (9)	0.0863 (10)	0.0078 (7)	0.0171 (8)	0.0277 (7)
N1	0.0377 (7)	0.0774 (10)	0.0562 (8)	0.0055 (7)	0.0054 (6)	0.0268 (7)
N2	0.0354 (6)	0.0546 (8)	0.0422 (7)	0.0024 (6)	0.0075 (5)	0.0110 (6)
N3	0.0592 (8)	0.0581 (9)	0.0484 (8)	−0.0008 (7)	0.0160 (7)	0.0056 (7)
N4	0.0494 (7)	0.0532 (8)	0.0432 (7)	−0.0058 (6)	0.0048 (6)	0.0056 (6)
N5	0.0649 (10)	0.0591 (9)	0.0535 (9)	−0.0065 (8)	0.0181 (7)	0.0079 (7)
C1	0.0691 (12)	0.0473 (10)	0.0758 (13)	0.0031 (9)	−0.0005 (10)	0.0118 (9)
C2	0.0747 (15)	0.0852 (16)	0.134 (2)	−0.0138 (13)	0.0032 (14)	0.0445 (16)
C3	0.156 (3)	0.0629 (15)	0.124 (2)	0.0019 (17)	0.011 (2)	−0.0182 (15)
C4	0.0856 (15)	0.0896 (16)	0.0818 (15)	0.0095 (13)	−0.0074 (12)	0.0244 (13)
C5	0.0421 (9)	0.0593 (10)	0.0487 (9)	0.0045 (8)	0.0001 (7)	0.0036 (8)
C6	0.0403 (8)	0.0773 (12)	0.0574 (10)	−0.0038 (8)	0.0026 (7)	0.0172 (9)
C7	0.0358 (8)	0.0628 (10)	0.0540 (9)	0.0031 (7)	0.0112 (7)	0.0118 (8)
C8	0.0333 (8)	0.0714 (11)	0.0573 (10)	0.0014 (8)	0.0060 (7)	0.0216 (8)
C9	0.0411 (8)	0.0762 (12)	0.0554 (10)	0.0142 (8)	0.0142 (7)	0.0245 (9)
C10	0.0463 (9)	0.0600 (10)	0.0444 (9)	−0.0030 (8)	0.0053 (7)	0.0093 (7)
C11	0.0469 (8)	0.0537 (9)	0.0378 (8)	−0.0015 (7)	0.0033 (6)	0.0070 (7)
C12	0.0597 (10)	0.0529 (10)	0.0454 (9)	0.0014 (8)	0.0122 (7)	0.0069 (7)
C13	0.0506 (9)	0.0558 (10)	0.0385 (8)	−0.0084 (8)	0.0047 (7)	0.0028 (7)
C14	0.0428 (8)	0.0546 (9)	0.0377 (8)	−0.0033 (7)	−0.0005 (6)	0.0035 (7)
C15	0.0475 (9)	0.0512 (9)	0.0500 (9)	−0.0018 (7)	0.0027 (7)	0.0020 (7)
C16	0.0683 (12)	0.0707 (12)	0.0607 (11)	0.0132 (10)	0.0153 (9)	0.0075 (9)
C17	0.0839 (15)	0.0805 (15)	0.0813 (15)	0.0171 (12)	0.0316 (12)	0.0045 (12)
C18	0.0726 (13)	0.0613 (13)	0.1083 (18)	0.0137 (11)	0.0234 (13)	−0.0116 (12)
C19	0.0693 (12)	0.0494 (11)	0.0952 (16)	0.0089 (9)	0.0092 (11)	0.0029 (10)
C20	0.0567 (10)	0.0460 (9)	0.0672 (11)	−0.0044 (8)	0.0020 (8)	0.0042 (8)

### Geometric parameters (Å, º)

**Table:**

O1—C5	1.336 (2)	C4—H4C	0.9600
O1—C1	1.464 (2)	C6—C7	1.504 (2)
O2—C5	1.2080 (19)	C6—H6A	0.9700
O3—C20	1.354 (2)	C6—H6B	0.9700
O3—H1O	0.832 (10)	C7—H7A	0.9700
N1—C5	1.350 (2)	C7—H7B	0.9700
N1—C6	1.447 (2)	C8—C9	1.505 (2)
N1—C9	1.453 (2)	C8—H8A	0.9700
N2—C7	1.4513 (19)	C8—H8B	0.9700
N2—C8	1.4564 (19)	C9—H9A	0.9700
N2—C10	1.467 (2)	C9—H9B	0.9700
N3—C12	1.326 (2)	C10—C11	1.503 (2)
N3—C13	1.345 (2)	C10—H10A	0.9700
N4—C13	1.338 (2)	C10—H10B	0.9700
N4—C14	1.3476 (19)	C11—C12	1.378 (2)
N5—C13	1.339 (2)	C11—C14	1.403 (2)
N5—H1N	0.861 (9)	C12—H12	0.9300
N5—H2N	0.859 (9)	C14—C15	1.477 (2)
C1—C4	1.502 (3)	C15—C16	1.396 (2)
C1—C3	1.500 (3)	C15—C20	1.411 (2)
C1—C2	1.513 (3)	C16—C17	1.371 (3)
C2—H2A	0.9600	C16—H16	0.9300
C2—H2B	0.9600	C17—C18	1.375 (3)
C2—H2C	0.9600	C17—H17	0.9300
C3—H3A	0.9600	C18—C19	1.362 (3)
C3—H3B	0.9600	C18—H18	0.9300
C3—H3C	0.9600	C19—C20	1.375 (3)
C4—H4A	0.9600	C19—H19	0.9300
C4—H4B	0.9600		
			
C5—O1—C1	121.59 (13)	C6—C7—H7B	109.5
C20—O3—H1O	106 (2)	H7A—C7—H7B	108.1
C5—N1—C6	120.09 (14)	N2—C8—C9	111.36 (14)
C5—N1—C9	124.84 (14)	N2—C8—H8A	109.4
C6—N1—C9	113.22 (13)	C9—C8—H8A	109.4
C7—N2—C8	109.85 (12)	N2—C8—H8B	109.4
C7—N2—C10	112.32 (12)	C9—C8—H8B	109.4
C8—N2—C10	109.84 (12)	H8A—C8—H8B	108.0
C12—N3—C13	114.41 (15)	N1—C9—C8	109.68 (13)
C13—N4—C14	119.56 (14)	N1—C9—H9A	109.7
C13—N5—H1N	116.6 (14)	C8—C9—H9A	109.7
C13—N5—H2N	115.1 (14)	N1—C9—H9B	109.7
H1N—N5—H2N	128 (2)	C8—C9—H9B	109.7
O1—C1—C4	110.10 (16)	H9A—C9—H9B	108.2
O1—C1—C3	110.25 (18)	N2—C10—C11	114.62 (13)
C4—C1—C3	111.4 (2)	N2—C10—H10A	108.6
O1—C1—C2	101.62 (15)	C11—C10—H10A	108.6
C4—C1—C2	110.11 (19)	N2—C10—H10B	108.6
C3—C1—C2	112.9 (2)	C11—C10—H10B	108.6
C1—C2—H2A	109.5	H10A—C10—H10B	107.6
C1—C2—H2B	109.5	C12—C11—C14	115.08 (14)
H2A—C2—H2B	109.5	C12—C11—C10	117.72 (15)
C1—C2—H2C	109.5	C14—C11—C10	126.96 (15)
H2A—C2—H2C	109.5	N3—C12—C11	126.47 (17)
H2B—C2—H2C	109.5	N3—C12—H12	116.8
C1—C3—H3A	109.5	C11—C12—H12	116.8
C1—C3—H3B	109.5	N4—C13—N5	118.34 (16)
H3A—C3—H3B	109.5	N4—C13—N3	124.67 (14)
C1—C3—H3C	109.5	N5—C13—N3	116.98 (15)
H3A—C3—H3C	109.5	N4—C14—C11	119.68 (14)
H3B—C3—H3C	109.5	N4—C14—C15	114.77 (14)
C1—C4—H4A	109.5	C11—C14—C15	125.46 (14)
C1—C4—H4B	109.5	C16—C15—C20	116.87 (16)
H4A—C4—H4B	109.5	C16—C15—C14	121.81 (15)
C1—C4—H4C	109.5	C20—C15—C14	121.32 (15)
H4A—C4—H4C	109.5	C17—C16—C15	122.15 (19)
H4B—C4—H4C	109.5	C17—C16—H16	118.9
O2—C5—O1	125.84 (16)	C15—C16—H16	118.9
O2—C5—N1	123.43 (16)	C16—C17—C18	119.2 (2)
O1—C5—N1	110.66 (13)	C16—C17—H17	120.4
N1—C6—C7	110.82 (14)	C18—C17—H17	120.4
N1—C6—H6A	109.5	C19—C18—C17	120.32 (19)
C7—C6—H6A	109.5	C19—C18—H18	119.8
N1—C6—H6B	109.5	C17—C18—H18	119.8
C7—C6—H6B	109.5	C18—C19—C20	121.1 (2)
H6A—C6—H6B	108.1	C18—C19—H19	119.5
N2—C7—C6	110.82 (13)	C20—C19—H19	119.4
N2—C7—H7A	109.5	O3—C20—C19	117.65 (17)
C6—C7—H7A	109.5	O3—C20—C15	122.34 (16)
N2—C7—H7B	109.5	C19—C20—C15	119.98 (18)
			
C5—O1—C1—C4	63.7 (2)	C14—N4—C13—N5	179.31 (15)
C5—O1—C1—C3	−59.6 (2)	C14—N4—C13—N3	0.6 (2)
C5—O1—C1—C2	−179.57 (18)	C12—N3—C13—N4	2.4 (2)
C1—O1—C5—O2	−3.9 (3)	C12—N3—C13—N5	−176.31 (16)
C1—O1—C5—N1	178.86 (16)	C13—N4—C14—C11	−3.2 (2)
C6—N1—C5—O2	5.5 (3)	C13—N4—C14—C15	−179.82 (14)
C9—N1—C5—O2	168.93 (18)	C12—C11—C14—N4	2.5 (2)
C6—N1—C5—O1	−177.24 (15)	C10—C11—C14—N4	−171.82 (15)
C9—N1—C5—O1	−13.8 (2)	C12—C11—C14—C15	178.75 (15)
C5—N1—C6—C7	111.09 (18)	C10—C11—C14—C15	4.5 (3)
C9—N1—C6—C7	−54.2 (2)	N4—C14—C15—C16	−157.67 (17)
C8—N2—C7—C6	−58.01 (19)	C11—C14—C15—C16	25.9 (3)
C10—N2—C7—C6	179.42 (14)	N4—C14—C15—C20	23.2 (2)
N1—C6—C7—N2	55.5 (2)	C11—C14—C15—C20	−153.26 (16)
C7—N2—C8—C9	58.83 (18)	C20—C15—C16—C17	4.9 (3)
C10—N2—C8—C9	−177.15 (14)	C14—C15—C16—C17	−174.32 (19)
C5—N1—C9—C8	−110.40 (19)	C15—C16—C17—C18	0.1 (4)
C6—N1—C9—C8	54.0 (2)	C16—C17—C18—C19	−3.6 (4)
N2—C8—C9—N1	−56.0 (2)	C17—C18—C19—C20	1.9 (4)
C7—N2—C10—C11	−59.36 (19)	C18—C19—C20—O3	−178.7 (2)
C8—N2—C10—C11	178.07 (14)	C18—C19—C20—C15	3.3 (3)
N2—C10—C11—C12	98.42 (18)	C16—C15—C20—O3	175.57 (18)
N2—C10—C11—C14	−87.4 (2)	C14—C15—C20—O3	−5.2 (3)
C13—N3—C12—C11	−3.1 (3)	C16—C15—C20—C19	−6.5 (3)
C14—C11—C12—N3	0.8 (3)	C14—C15—C20—C19	172.72 (17)
C10—C11—C12—N3	175.61 (16)		

### Hydrogen-bond geometry (Å, º)

**Table:**

*D*—H···*A*	*D*—H	H···*A*	*D*···*A*	*D*—H···*A*
O3—H1*O*···N4	0.83 (1)	1.80 (2)	2.560 (2)	151 (3)
N5—H1*N*···N3^i^	0.86 (1)	2.19 (1)	3.054 (2)	179 (2)
N5—H2*N*···O3^ii^	0.86 (1)	2.29 (1)	3.139 (2)	169 (2)

Symmetry codes: (i) −*x*+3, −*y*+1, −*z*; (ii) −*x*+2, −*y*+2, −*z*.
